# Role of age in dynamics of autoantibodies in pediatric Celiac disease

**DOI:** 10.1186/s13052-023-01435-6

**Published:** 2023-03-23

**Authors:** Chiara Maria Trovato, Monica Montuori, Beatrice Leter, Ilaria Laudadio, Giusy Russo, Salvatore Oliva

**Affiliations:** 1 Gastroenterology and Nutritional Rehabilitation Unit, I.R.C.C.S. Bambino Gesù Children’s Hospital, Piazza Sant’Onofrio 4, 00165 Rome, Italy; 2 Pediatric Gastroenterology and Liver Unit, Maternal and Child Health Department, Sapienza University of Rome, Rome, Italy; 3 Department of Molecular Medicine, Sapienza University of Rome, Rome, Italy

**Keywords:** Anti-tissue transglutaminase, Anti-endomysial, Celiac disease, Children

## Abstract

**Background:**

Celiac disease (CD) is characterized by elevated serum titers of autoantibodies IgA anti-tissue transglutaminase 2 (TGA-IgA) and IgA anti-endomysial (EMA), with small bowel mucosa atrophy. We evaluated age differences between CD children exhibiting variable antibody titers at diagnosis.

**Methods:**

CD children diagnosed between January 2014 and June 2019, according to 2012 ESPGHAN guidelines were studied. All had EMA and TGA-IgA measurements, while a proportion of them underwent esophagogastroduodenoscopy (EGD). Patients were grouped based on serum TGA-IgA titers normalized to the upper limit of normal (ULN) and differences in median age (years) assessed by analysis of variance (ANOVA) and creation of orthogonal contrasts.

**Results:**

CD was diagnosed in 295 subjects (median age: 4.4 [IQR: 2.60–8.52]) with a biopsy sparing protocol (high titer: ≥ 10xULN) and in 204 by EGD biopsy. Of the latter, 142 (median age: 8.5 [IQR: 5.81–11.06]) and 62 (median age: 9.5 [IQR: 6.26–12.76]) had a low (< 5xULN) and a moderate (≥ 5 < 10xULN) TGA-IgA titer, respectively. Potential CD was diagnosed in 20 patients (median age: 3.6 [IQR: 2.47–6.91]). The median age was significantly lower in the no-biopsy group (ANOVA: F_(3, 516)_ = 25.98, *p* < .001) than in low- and moderate titer groups (*p* < 0.0001), while there was no statistical difference between biopsy-sparing and potential CD groups.

**Conclusion:**

CD patients with greatly elevated antibody titers (≥ 10xULN) were diagnosed at an earlier age than those with lower titers. This may indicate that an increase in TGA-IgA is independent of age and suggests a polarization of autoimmunity in younger individuals with higher serum antibody levels.

## Introduction

Celiac disease (CD) is one of the most common autoimmune diseases worldwide, caused by gluten in genetically susceptible individuals, and presents with a variety of signs and symptoms. Like all autoimmune diseases, it can affect people of all ages, including the elderly [1, 2]. The prevalence of CD has increased dramatically in recent decades, mainly in pediatric age [3]. Characteristically, CD patients have highly specific serum autoantibodies against the major CD autoantigen, tissue transglutaminase 2 (TG2) and against circulating deamidated gliadin peptides (DGP), as well as varying degrees of small intestinal mucosa atrophy [4].

Several reports highlight the positive association between serum levels of IgA anti tissue transglutaminase 2 (TGA-IgA) and the degree of mucosal villous atrophy [5, 6]. Therefore, the latest guidelines from the European Society of Pediatric Gastroenterology, Hepatology and Nutrition (ESPGHAN), allow the diagnosis of CD without biopsies in children with serum TGA-IgA 10 times or more the upper limit of normal (≥ 10xULN), confirmed by detection of IgA endomysial antibodies (EMA-IgA) in a second blood sample [7, 8]. In addition, these guidelines also recommend combining total IgA and TGA-IgA as an initial test, regardless of age.

Different types of autoantibodies can be used in CD diagnosis, but serum TGA-IgA are the most suitable for screening purposes because of their high sensitivity and specificity. Anti-transglutaminase seroconversion can stably occur around 21 months of age [9]: before this time, an additional autoantibody, such as DGP-IgG, is usually suggested for diagnosis [10]. In patients with non-elevated titers, duodenal-jejunal biopsies are critical for CD diagnosis.

There is still no clear evidence regarding the occurrence of seroconversion. To date, a few studies [11, 12] have examined age differences at diagnosis in CD children with different antibody titers. Therefore, in this study we aimed to characterize age differences between groups of CD children with different serum antibody titers.

## Methods

### Study population

We retrospectively enrolled all patients referred to the Pediatric Gastroenterology and Hepatology Unit at the Sapienza University Hospital Umberto I in Rome with suspected CD, between January 2014 and September 2019. Exclusion criteria included IgA deficiency and other chronic intestinal disorders such as food allergies, inflammatory bowel disease, infectious and immunological diseases, systemic disorders affecting the gut. All subjects were diagnosed according to the ESPGHAN criteria published in 2012 [7].

Patients with serum TGA-IgA < 10xULN underwent esophagogastroduodenoscopy (EGD) with multiple duodenum-jejunal biopsies, under general anesthesia or deep sedation [13]. Histological lesions were graded according to criteria of Marsh–Oberhuber (MO) [14]. TGA-IgA antibody titers were tested using commercially available ELISA kits from Eurospital (Trieste, Italy; cutoff value > 9 UA/mL).

The study population was divided into groups for analysis and comparison according to the need for biopsies, mean antibody titers, and the presence or absence of duodenal mucosa atrophy, as suggested in a previously published cohort [15].

- Biopsy-sparing group: patients with TGA-IgA ≥ 10 ULN, serum EMA IgA positivity, genetic predisposition (presence of HLA DQ2/HLA DQ8) and presence of symptoms and signs associated with CD (diarrhea, weight loss, failure to thrive, anorexia, abdominal distention, abdominal pain, short stature, flatulence, irritability, elevated titers of liver enzymes, constipation, and anemia) diagnosed without EGD and biopsy, per ESPGAHN 2012 guidelines.
- Patients requiring EGD with biopsy: a) symptomatic or asymptomatic children with serum TGA-IgA ≥ 5 < 10xULN, and mucosal lesions consistent with CD at biopsy were classified as moderate titer CD (at least two prior measurements of serum TGA-IgA were consistently in this range); b) symptomatic or asymptomatic children with serum levels of TGA-IgA < 5xULN and mucosal damage compatible with CD at biopsy (at least two prior measurements of TGA-IgA were consistently in this range), defined as low titer CD.
- Potential CD: symptomatic children with positive autoantibodies (TGA-IgA and at least one EMA detection positive), and with normal mucosa biopsies at various sites along the duodenum.

### Statistical analysis

Statistical analysis was performed using the R statistical software (R Core Team, 2019, version 3.6.1), using *the tidy verse package* [16] for data cleaning and presentation.

Differences between the median age of the groups were examined by analysis of variance (ANOVA), through the creation of orthogonal contrasts, with a significance of 0.05 for all statistical tests.

## Results

**Fig. 1:**
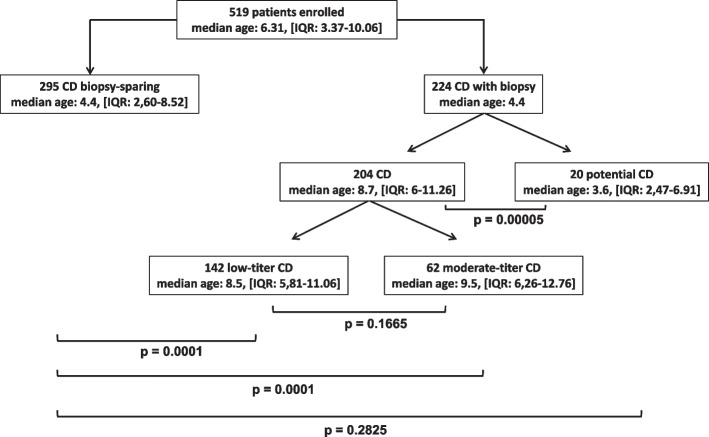
Patients investigated grouped according to the diagnostic protocol (biopsy sparing and endoscopic biopsy). For each group are reported median age (years) and interquartile ranges

Of 1243 children referred to our Unit with suspected CD during the study period, 519 patients (median age: 6.31 years [IQR: 3.37–10.06]; 335 females) were retrospectively enrolled. Population data are summarized in Fig. 1. Of these, 295 were included in the biopsy-sparing group (median age: 4.4 [IQR: 2.60–8.52]; 196 females), 62 in the moderate titer group (median age: 9.5 [IQR: 6.26–12.76]; 38 females), 142 in the low-titer group (median age: 8.5 [IQR: 5.81–11.06]; 86 females) and finally 20 in the potential CD group (median age: 3.6 [IQR: 2.47–6.91]; 13 females). The characteristics of the patient population are summarized in the Table 1.

**Table 1:** Characteristics of the Study Participants

Groups of patients	Biopsy-sparing	Low titer CD	Moderate titer CD	Potential CD
**Number of patients**	295	142	62	20
**Males**	99	56	24	7
**Females**	196	86	38	13
**Age, median [IQR]**	4.4 [2.60–8.52]	8.5 [5.81–11.06]	9.5 [6.26–12.76]	3.6 [2.47–6.91]
**Median TGA-IgA** (**ULN)**	> 10	2.2	9.03	1.81
**EMA positive (n. of patients)**	295	113	60	15
**EMA negative (n. of patients)**	0	29	2	5

*Legend*: *CD* Celiac disease, *TGA-IgA* autoantibodies IgA anti-tissue transglutaminase 2, *IQR* interquartile ranges, *ULN* upper limit of normal, *EMA* IgA anti-endomysial

**Fig. 2:**
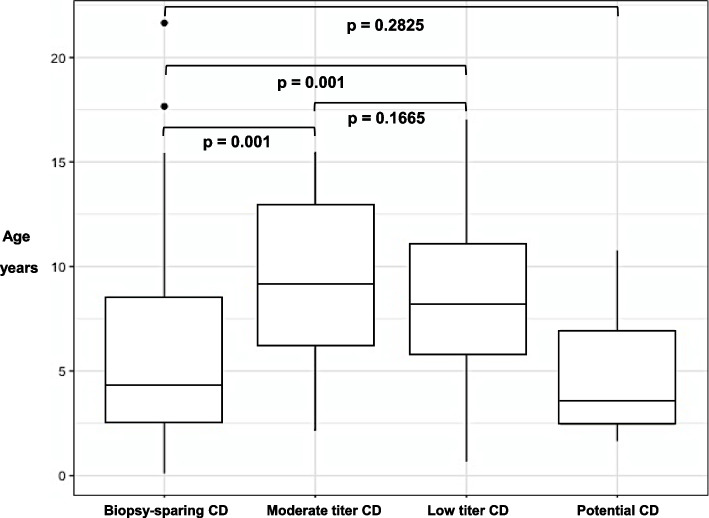
Graphic representation of median ages (years) and interquartile ranges for different groups of patients

Figure 2 shows a graphical representation of the median ages of the different groups. The age of patients in the biopsy-sparing group was significantly lower (ANOVA: F_(3, 516)_ = 25.98, *p* < 0.001) than in the low (*p* < 0.0001) and moderate (*p* < 0.0001) titer groups. No statistically significant difference was observed between the median ages of the biopsy-sparing group and the potential CD patients (*p* = 0.2825); moreover, no difference was found between low and moderate titer CD groups (8.5 [IQR: 5.81–11.06] vs 9.5 [IQR: 6.26–12.76], respectively; *p* = 0.1665).

As shown in the Table 1, it is worth noting that 29 and 2 of the low and moderate titers, respectively, received a diagnosis of CD despite negative EMA-IgA serum levels. Of the PCD group, 5 children were EMA negative when they underwent EGD, but all 20 PCD children had at least one EMA positive test.

## Discussion

The present study, carried out on several groups of pediatric patients with CD, was aimed to analyze the age differences at diagnosis in relation to different specific autoantibody titers. We found that children diagnosed using the “biopsy-sparing protocol”, with the highest serum TGA-IgA titers (≥ 10xULN), were significantly younger than the other CD groups.

To date there is still little data focusing on the association between the age at presentation of children with CD and titers of serum specific autoantibodies. Our study confirms previous results showing that CD children tend to have high TGA-IgA titers, particularly at a younger age [11]. The dynamics of autoantibody development are still unclear, and it is unclear why the levels of autoantibody markers are so different in the pediatric CD population. In 2005, Salmi et al. [17] found lower titers of TGA-IgA autoantibodies in EMA-negative adult CD patients: the authors suggested a possible entrapment of autoantibodies in the intestinal mucosa which would prevent them from entering the blood, due to a higher tissue avidity of autoantibodies in a long-standing disease.

Interestingly, our data on higher titers of TGA-IgA autoantibodies in the youngest children appear to parallel the study by Marine et al. [3] showing for the first time that children, mainly the youngest, have a higher CD prevalence compared to adults. We are tempted to speculate that the difference in serum autoantibody titers might be due to a polarization of the immune system towards overproduction of TGA-IgA autoantibodies, in parallel with a concomitant reduced immunological tolerance. As is known, upon recognition of foreign antigenic peptides presented by MHC native T cells are activated and clonally expanded. Based on a recent study by Yao et al. [18], we believe that different types of lymphocytes could be activated by differential clonal expansion as well as autoantibody production. In the same way, cytokine expression could modulate the production of autoantibodies since they can selectively affect intraepithelial cytotoxic T cells [19].

However, this specific mechanism needs to be studied in detail before this hypothesis can be confirmed. The high prevalence of seronegative CD in adults [15, 20] supports our speculation.

The relationship between age and autoantibodies at onset in other autoimmune diseases has been studied. In fact, as reported in type 1 diabetes [21], children with an early age at onset of the disease have higher levels of autoantibodies and more autoimmune diseases. Interestingly, among children at high genetic risk for type 1 diabetes, those with late onset islet autoimmunity tend to develop diabetes in adolescence or early adulthood [21, 22]; in addition, there is widespread agreement that in various autoimmune diseases (e.g. systemic lupus erythematosus, type 1 diabetes mellitus) an early age at onset can act as a negative prognostic factor for the course of the disease [23]. Interestingly, data from studies of our group of CD patients suggest that age at diagnosis is a strong predictor for the occurrence of organ-specific autoantibodies and the development of additional autoimmune diseases [24]. We cannot determine whether the dynamics of other autoimmune diseases would differ between CD children who underwent EGD and those with the highest levels of serum autoantibodies diagnosed by a biopsy-sparing protocol. However, this could be an important research topic to fully understand autoantibody’ “autonomy” with respect to other autoimmune diseases.

Interestingly, we observed no statistical difference in patient age (*p* = 0.1665) between the low and the moderate titer groups: this may indicate that autoantibodies in a large proportion of subjects do not continuously increase over time. This could also bring further focus to clinical scenarios with positive low-to-moderate serum TGA-IgA levels, for which a clear and linear diagnostic work-up is not yet defined by guidelines [25].

Notably, potential CD children were significantly younger than those with biopsy-proven histological defects. This finding can be explained by the “progression of mucosal damage” [26]: indeed, due to the patchy damage to the small bowel mucosa in CD [27], it is conceivable that the damaged areas are not initially identified in some children. However, as the number of “damaged areas” increases over time, these patients can later be identified as “overt CD patients”, despite non-elevated autoantibody titers. Undoubtedly, age is one of the variables to consider when assessing the risk of progression from potential to overt CD [28]: since potential CD patients appear to be younger, a rigorous follow-up is remarkably important to intercept the potential transtition to overt CD [29].

It is worth noting that the median age was not statistically different between the biopsy-sparing and the potential CD groups, suggesting that, in autoimmunity, the peak of autoantibodies would resemble a sudden, uncontrolled storm manifesting unexpectedly and with an unpredictable autoantibody titer.

## Conclusions

In summary, although our study has some limitations such as the lack of an adult control group and the small sample size enrolled by an academic tertiary care center, two groups of CD children were identified: one is characterized by individuals who quickly reach elevated autoantibody titers (with levels ≥ 10 times the upper limit of normal); the second includes subjects who produce less circulating autoantibodies and never reach high titers. The relevance of these results remains an open question for pediatric gastroenterologists. Further studies will focus on deciphering the immunological signaling pathways beyond these observations as well as understanding the dynamics of specific autoantibodies in CD children with different serum titers. In addition, the inclusion of an adult CD population would be worthwhile.

## Data Availability

The datasets used and analyzed during the current study are available from the corresponding author on reasonable request.

## Abbreviations

CD: Celiac disease
TGA-IgA: IgA anti-tissue transglutaminase 2
EMA: And IgA anti-endomysial
EGD: Esophagogastroduodenoscopy
ULN: Upper limit of normal
ESPGHAN: European Society of Pediatric Gastroenterology, Hepatology and Nutrition
